# Therapeutic Effects of hMAPC and hMSC Transplantation after Stroke in Mice

**DOI:** 10.1371/journal.pone.0043683

**Published:** 2012-08-31

**Authors:** Silvia Mora-Lee, Mª Salomé Sirerol-Piquer, María Gutiérrez-Pérez, Ulises Gomez-Pinedo, Valerie D. Roobrouck, Tania López, Mayte Casado-Nieto, Gloria Abizanda, Maria Teresa Rabena, Catherine Verfaille, Felipe Prósper, Jose Manuel García-Verdugo

**Affiliations:** 1 Hematology and Cell Therapy Area, Clinica Universidad de Navarra and Division of Cancer, Center for Applied Medical Research, University of Navarra, Pamplona, Spain; 2 Department of Comparative Neurobiology, Cavanilles Institute of Biodiversity and Evolutive Biology, University of Valencia, Centro de Investigación Biomédica en Red sobre Enfermedades Neurodegenerativas, Valencia, Spain; 3 Laboratorio de Medicina Regenerativa, Hospital Clínico San Carlos, Madrid, Madrid, Spain; 4 Stem Cell Institute, K. U. Leuven, Belgium; 5 Department of Statistics, University of Valencia, Valencia, Spain; Université Pierre et Marie Curie-Paris6, INSERM, CNRS, France

## Abstract

Stroke represents an attractive target for stem cell therapy. Although different types of cells have been employed in animal models, a direct comparison between cell sources has not been performed. The aim of our study was to assess the effect of human multipotent adult progenitor cells (hMAPCs) and human mesenchymal stem cells (hMSCs) on endogenous neurogenesis, angiogenesis and inflammation following stroke. BALB/Ca-RAG 2^−/−^ γC^−/−^ mice subjected to FeCl_3_ thrombosis mediated stroke were intracranially injected with 2×10^5^ hMAPCs or hMSCs 2 days after stroke and followed for up to 28 days. We could not detect long-term engraftment of either cell population. However, in comparison with PBS-treated animals, hMSC and hMAPC grafted animals demonstrated significantly decreased loss of brain tissue. This was associated with increased angiogenesis, diminished inflammation and a glial-scar inhibitory effect. Moreover, enhanced proliferation of cells in the subventricular zone (SVZ) and survival of newly generated neuroblasts was observed. Interestingly, these neuroprotective effects were more pronounced in the group of animals treated with hMAPCs in comparison with hMSCs. Our results establish cell therapy with hMAPCs and hMSCs as a promising strategy for the treatment of stroke.

## Introduction

The use of cell therapy for the treatment of neurological disorders is currently an area of intense research. Stroke represents a particularly attractive target being the third leading cause of death and disability in western countries. There is a lack of successful therapies and only the use of recombinant tissue plasminogen activator has demonstrated some efficacy, which is actually restricted by the narrow window of therapeutic efficacy. Critical to the success of cell therapies is the selection and mode of delivery of cell populations. Various cell types including neural stem cells, endothelial precursor cells, cord blood cells and mesenchymal stem cells (MSC) have been tested as therapeutic agents, providing compelling evidence that application of stem/progenitor cells is safe and effective in animal models of stroke [1], [2], [3], [4], [5], [6]. However, before embarking on clinical trials in stroke patients a number of issues need to be addressed [7].

Similar to what has been observed in other ischemic diseases such as myocardial infarction, the hope that stem/progenitor cells could contribute to the restoration of the damaged tissue seems currently unrealistic [8], [9]. The more likely explanation for the beneficial effects observed in animal models is that the transplanted cells provide the damaged tissue with an improved microenvironment through the secretion of cytokines and trophic factors (VEGF, BDNF, bFGF, NGF…) [2], [10], [11], [12], [13]. Such molecules may reduce apoptotic cell death, promote angiogenesis, attenuate inflammation, reduce glial-scar formation and activate endogenous brain remodeling [1], [11], [14], [15], [16], [17].

From all the cell types tested, MSCs have emerged as one of the leading candidate populations in stroke management, as they are easily obtained from a range of tissues (including bone marrow, fat and other somatic tissues [18]), can be rapidly expanded in vitro while maintaining their potential to differentiate into multiple tissues [19], [20], [21]. An additional advantage is that MSCs are immune-privileged and thus could be explored in the allogeneic setting with a reduced risk of rejection. In fact a limited number of clinical studies have safely transplanted human MSCs in patients with stroke with promising results [22], [23], [24].

Recently, multipotent adult progenitor cells (MAPCs), a subpopulation of stem cells isolated from bone marrow, have been described and characterized. Human MAPCs are multipotent stem cells that have been shown to differentiate into various mesodermal cell types, with a remarkable proliferative capacity in culture [25]. In particular, their vascular potential *in vitro* and *in vivo* has been demonstrated which make them an attractive candidate for novel cell-based treatment of ischemic diseases. Moreover MAPCs are also immune-privileged [26], [27], [28], [29], [30].

The current study was designed to examine whether ischemic brain injury can be ameliorated by hMAPC administration, in comparison with hMSC transplantation in a thrombosis middle cerebral artery occlusion (MCAo) model.

## Materials and Methods

### Preparation of Cells

hMSCs clone JN1 and hMAPC clone B30/E2 were provided in frozen vials by Athersys, Inc. Cells were prepared under GMP (good manufacturing practice) as previously described [31]. At the time of transplantation, cells were thawed, resuspended in PBS and cell density was adjusted to a final concentration of 1×10^5^ cells/3.0 µl.

For fluorescence-activated cell sorting (FACS) analysis, cells were detached with 0.05% trypsin-EDTA and washed with PBS. hMAPCs and hMSCs ranging in number between 1×10^5^ to 2×10^5^ hMSCs were incubated with fluorescent conjugated primary antibodies for 15 min in the dark at room temperature (RT). The following antibodies were used: CD44-FITC, CD45-FITC, HLA-II-PE, CD144-FITC, CD34-FITC, HLA-I-APC, CD105-APC, CD140a-PE, CD90-APC, CD73-PE, CD117-PE, and CD31-PE, and their corresponding isotype controls (all from BD Pharmingen). Cells were fixed with 4% paraformaldehyde (PFA) at 4°C (Figure S1) (Roobrouck et al., 2011).

### Animals and Surgical Procedures

All experiments were performed in accordance with the “Principles of Laboratory Animal Care”, and all procedures involving animals were approved by the Animal Ethical Committee of our Institute (University of Navarra Institutional Committee on the Care and Use of Laboratory Animals). Mice were kindly provided by Dr. Mamoru (from the Central Institute for Experimental Animals, Kawasaki, Japan), bred and housed in a temperature-controlled room with access to food and water ad libitum Thrombotic stroke was induced in adult (7-week-old) male BALB/Ca-RAG2^−/−^γc^−/−^ mice under controlled conditions.

#### Ferric chloride thrombosis stroke model

Mice were anesthetized with 80–100 mg/kg i.p ketamine hydrochloride and 10-mg/kg i.p xylazine. Animals were placed in the right lateral position, the skin between the right eye and the right ear was incised, and the temporal muscle was retracted. A small craniotomy was performed, the dura was excised, and the middle cerebral artery (MCA) was exposed. Thrombus was induced by topical application of a small strip of filter paper soaked with 20% Ferric Chloride (Sigma-Aldrich) with the adventitial surface of the vessel. After 1 min of exposure the filter paper was removed and the vessel was washed with saline. Soft tissues were allowed to fall back into place and the skin was sutured [32]; [33], [34]. The sham group underwent similar operative procedure except that 0.9% saline was used instead of 20% ferric chloride. Animals received analgesia (Ketoprofen 5 mg/Kg) on the day of the surgery and each day thereafter, as well as antibiotic treatment (Enrofloxacin 25 mg/Kg) for 5 days.

#### Cell transplantation

After stroke, mice were randomly assigned to the treatment groups. Stereotaxic surgery was performed under anesthesia 2 days after stroke for mice recovery from previous stroke surgery and regarding potential human transplantation. Mice were given two 3.0 µl injections of hMSCs or hMAPCs or PBS (1×10^5^ cells/3 µl; or 3.0 µl of PBS) along the anterior-posterior axis at the following coordinates: 1) A–P:1.18; M–L:1.5; D–V:2.5; 2) A–P:−1.0; M–L:2.5; D–V: 2.6 [35]. A micro-infusion pump was used to control the speed of delivery at 0.5 µl/min. The needle was left *in situ* for 10 min post-injection before being slowly removed.

All the groups and a sham, which was not subjected to stroke and cell transplantation, were analyzed at different time points after cell transplantation (2 days, 4 days, 7 days and 28 days) (n = 7 per group and time point: n = 4 for immunohistochemical analysis and n = 3 for semithin section analysis; n = 28 animals per group) (Figure S2).

### Immunohistochemistry and Morphometric Analysis

At sacrifice, mice were intracardially perfused with PBS followed by 4% PFA. Brains were removed, postfixed overnight in 4% PFA and cryoprotected in 15% sucrose overnight (ON) at 4°C. Subsequently, brains were frozen and cryostat-sectioned coronally at 14 µm. Ten serial series (containing one section out of every 10) were prepared for volume analysis and immunohistochemical quantifications. Analyses were performed in a blinded fashion.

#### Measurement of cavitation

Tissue loss volume was measured in 4 mice per group 4 weeks after 28 days PBS or cell transplantation (Figure S2). At this time point necrotic tissue has been replaced by a cavity (or cyst), thereby dropping inflammation and edema [32]. One of each 10 serial sections per animal was thawed at RT for 20 min, washed with PBS and stained with 0.25% thionin (Sigma-Aldrich) at 60°C for 5 min. Then, sections were rinsed in water and dehydrated through graded alcohols, cleared in xylene and coverslipped in DPX mounting media (VWR Prolabo). The area of both hemispheres (mm^2^) was calculated by tracing the area on the computer screen. To reduce errors associated with processing of tissue for histological analysis, the area of cavitation in each section was presented as the percentage of the cavitation in comparison to the area of the contralateral hemisphere.

#### Immunofluorescence

For BrdU immunostaining, sections were pre-treated with 2N HCl (30 min at 37°C) and then rinsed for 15 min in 0.1M-borate buffer [pH 8.5]. For CD31 immunostaining, sections were pre-treated with 2% trypsin in 0.1% CaCl_2_ (7 min at 37°C). For NG2-Chondroitin Sulfate Proteoglycan (NG2-CSP) and fibronectin immunostaining sections were pre-treated with citrate buffer (20 min at 60°C). Sections were rinsed in PBS and incubated in blocking solution, followed by an ON incubation at 4°C with the primary antibody: mouse anti-BrdU, 1∶50 (DakoCytomation), mouse anti-human nuclei HuNu, 1∶100 (DakoCytomation), rat anti-CD31, 1∶500 (BD Biosciences), mouse anti-NG2-CSP, 1∶200 (Millipore), mouse anti-fibronectin, 1∶50 (Millipore) or goat anti-Doublecortin DCX, 1∶200 (Santa Cruz Biotechnology). Subsequently, sections were washed and incubated for 1 hr with the appropriate secondary antibody: mouse-Cy3 or rabbit-FITC, goat-Cy3, 1∶200 (Jackson Immunoresearch), or biotinylated rabbit anti-rat IgG antibody, 1∶300 (DakoCytomation). For CD31, the tyramide signal amplification kit, (TSA) (Perkin Elmer) was used according to the manufactureŕs instructions. The sections were mounted using vectashield with DAPI (Vector).

#### Immunohistochemistry

For microglia staining, sections were preincubated in endogenous peroxidase blocking solution, an incubated in blocking solution, followed by an overnight incubation at 4°C with rabbit anti-Iba1, 1∶200 (WAKO). The sections were then washed and incubated for 90 min with biotinylated goat anti-rabbit, 1∶250 (VECTOR) and then for 45 min with Avidin-Biotin-Peroxides Complex (ABC, 1∶250, VECTOR). The antibody staining was visualized with 0.05% DAB (Sigma) and sections were dehydrated and mounted with Eukit mounting media (VWR).

#### Neovascularization

To estimate the blood vessel density after stroke, five to seven CD31 stained, 14 µm coronal sections per animal (n = 4 in each group), spaced 140 µm apart were analyzed (Figure S2). Blood vessel density estimation was performed in the ischemic boundary region using an Axioplan 2-Zeiss automated microscope. This region was delineated from the edge of the pan-necrotic cystic cavity approximately 400 µm into the adjacent cortex. Within these boundaries four randomly chosen fields at 10x magnification were analyzed. The acquisition of the images was performed with a macro designed in Metamorph 6.3r6®. Blood vessel density was estimated as the percentage of CD31 positive area.

#### Inflammation

Microglia activation was evaluated by measuring shape transformation according to previously described criteria [36], [37]. A total of 25 microglial cells were randomly selected in a 300 µm wide area at the peri-infarct zone per section, in six coronal sections spaced 140 µm apart (150 microglial cells per animal) (n = 4 per group) 4 days after cell transplantation (Figure S2). The soma was placed in the center of a grid of 10 concentric circles, 5 µm apart and the number of intersections between cytoplasmic processes and grid lines was registered.

#### Scar-inhibition

For qualitative analysis, fibronectin and NG2-CSP two-dimensional pseudocolor images (4095 color levels) were gathered using Nikon C-HGFI fluorescence microscope. Quantification was assessed by measuring the percentage of threshold area occupied by NG2-CSP-positive or fibronectin staining in relation to the whole-tissue area in the peri-ischemic area. Approximately 8 fields of the ischemic cerebral cortex from five to eight coronal brain sections of four animals per group were analysed. Analysis was performed by optical density measurements (binary image processing of black and white), obtaining the percentage of NG2-CSP or fibronectin per visual field area analysed with the Image J 1.42 software (NIH).

#### SVZ proliferation

To evaluate the activation of SVZ proliferation, a single dose of BrdU (50 mg/kg) was injected 2 hours before sacrifice to mice in each group (n = 4; sham, PBS, hMSCs and hMAPCs), which were sacrificed at different time points after transplantation (2 days, 4 days, 7 days and 28 days). BrdU positive cells in the SVZ were counted blindly using five to seven 14 µm coronal sections per animal (n = 5–7), which were spaced 140 µm apart from one another (Figure S2). Cells were counted under high power on an Axio imager M1 Zeiss microscope. Results were expressed as the average number of BrdU-positive cells per section.

#### Endogenous neuroblast survival

Double labeling with HuNu and DCX (n = 4 in each group), in the peri-infarct 28 days after cell transplantation was performed (Figure S2).

For systematic random sampling in design-based stereological cell counting, four coronal brain sections per mouse were selected, spaced 140 µm apart across the same region of interest in each animal (from Bregma: +0.7 mm to − 0.7 mm). For multi-stage random sampling, 8 fields per brain section were randomly chosen in the ischemic border region under 40× magnifications on a confocal microscope. To estimate the DCX/DAPI index, DAPI-positive and DCX-positive cells were quantified in an area of 300 microns from the ischemic border region at the per-infarct zone 28 days after cell transplantation (the ischemic border was defined by histological characteristics, mainly by the presence of edematous tissue in the ischemic zone and the presence of polymorphonuclear stacked nuclei enclosing the affected neuropil at the glial scar, which at this time point is well established). This estimation consists in the number of DCX+ cells divided per the total amount of cells (counted by their nuclei, which is stained with DAPI). Quantification was performed under blind condition.

### Tissue Processing for Semithin Sections

Animals were perfused with PBS followed by 2% PFA and 2.5% glutaraldehyde. Brains were post-fixed overnight in the same fixative, cut into 200 µm coronal sections, and processed for semithin sections. 200 µm coronal sections were post-fixed in 2% osmium tetroxide for 2 hours, rinsed, dehydrated, and embedded in Durcupan (Fluka). To study time dependent activation of SVZ proliferation after cell transplantation, whole hemispheres obtained at different time points (2 days, 4 days, 7 days and 28 days) were cut into 1.5 µm semithin sections with a diamond knife and stained with 1% toluidine-blue.

### Statistical Analysis

Statistical significance was assessed by one-way or two-way analysis of variance (ANOVA) followed by the Bonferroni *post hoc* test using PASW Statistics 17.0 software®. A probability of P<0.05 was adopted for statistical significance. Data are reported as mean ± SD.

## Results

### Survival and Migration of Transplanted Cells after Intrastriatal Transplantation

BALB/Ca-RAG2^−/−^γc^−/−^ mice received an intracranial graft of 2×10^5^ hMAPCs or hMSCs 2 days after MCAo. Using antibodies against human nuclei, hMSCs and hMAPCs were clearly detected after 2 days in the transplant area. After 4 days both cell types were primarily concentrated at the transplantation site while migration of some cells towards the ischemic lesion was observed. However, 7 days after transplantation there was a decrease in the number of cells being detected both in the area of injection, as well as, in the ischemic lesion. No cells were detected at 28 days post-transplantation in the brain (Figure S3 and data not shown).

### Transplantation of hMAPCs and hMSCs is Associated with a Reduced Loss of Brain Tissue

**Figure 1:**
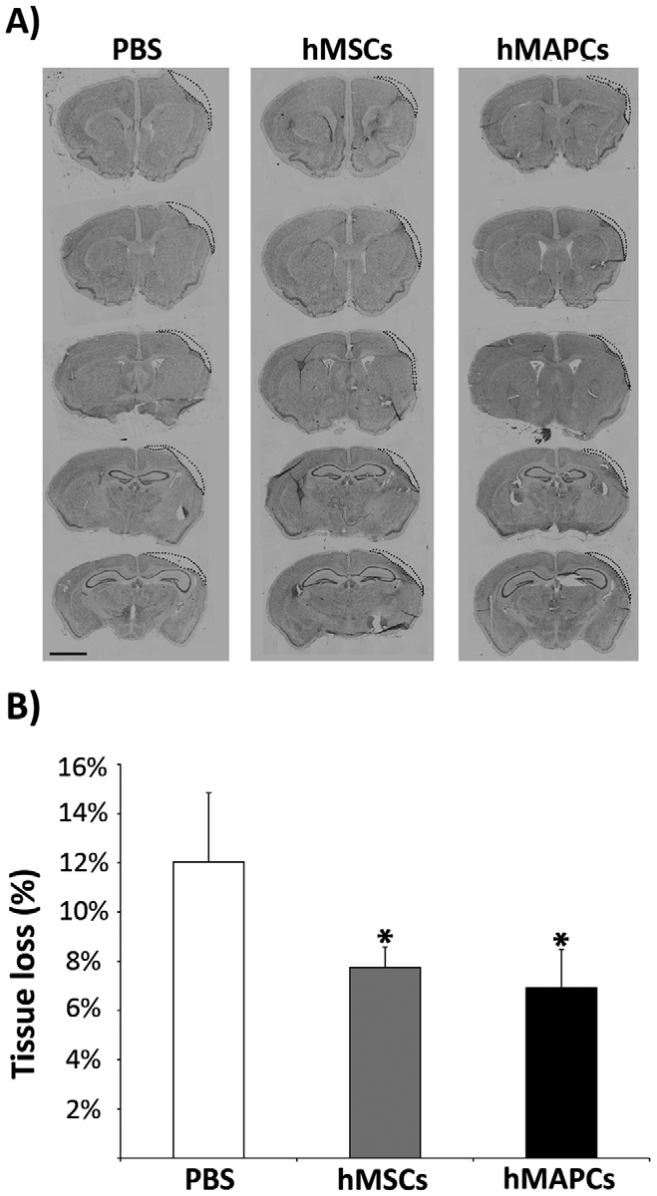
Effect of transplantation of hMSCs and hMAPCs in tissue loss. A) Nissl-staining photomicrographs of coronal sections that show the loss of tissue at 28 days after transplantation. The dashed line marks tissue loss. Scale bar = 1000 µm; B) Quantification of tissue loss 28 days after cell transplantation n = 4 *p<0.05 PBS-treated group vs cell transplanted groups by Bonferroni test.

Ischemic damage leads to loss of brain tissue and the development of various sizes of cysts in stroke mice (Fig. 1A). Interestingly, a significant decrease (*P*<0.05) in tissue loss was observed after 28 days in animals treated with hMSCs, as well as, hMAPCs (7.75%±0.8 and 6.9%±1.6, respectively) compared to the PBS-treated group of mice (12%±2.8%), suggesting a neuroprotective effect within the ischemic penumbra associated with cell transplantation (Fig. 1B).

### Cell Transplantation Induced an Increase in Angiogenesis after Stroke

Increased vascularization in the penumbra after stroke is associated with neurological recovery and offers another potential target for cell therapy [38], [39]. Because recent a number of studies have demonstrated that hMSC transplantation after stroke may induce angiogenesis [10], [11], [40], which may be related to the decrease in tissue damage, we decided to analyze blood vessel density.

**Figure 2:**
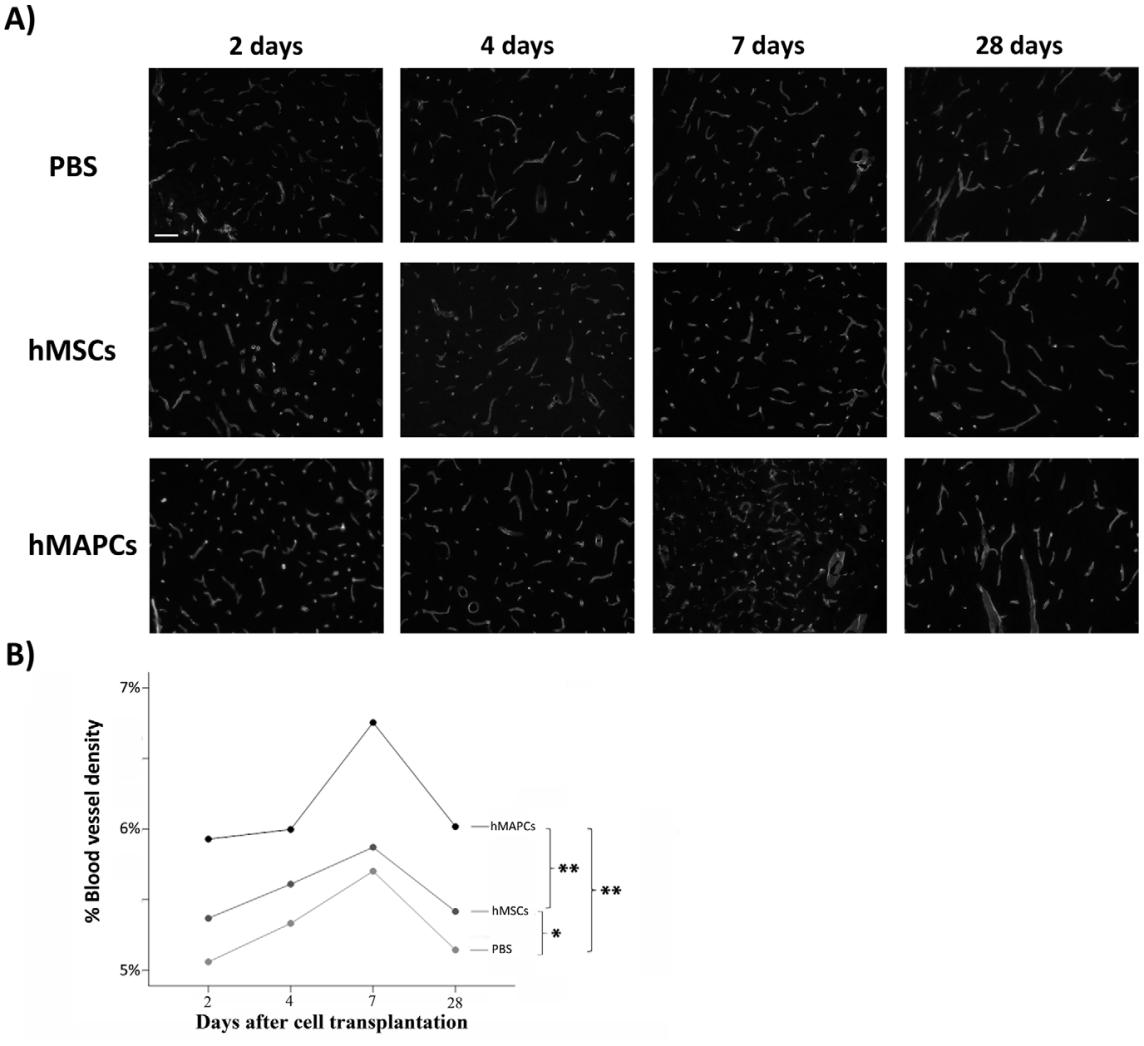
Transplantation of hMSCs and hMAPCs induced an increase in angiogenesis. A) CD31 immunostaining in the ischemic boundary zone at different time points after cell transplantation (2 days, 4 days, 7 days and 28 days) (Scale bar = 100 µm). B) Quantitative analysis of angiogenesis after cell transplantation (n = 4). *p<0.05 and ** *p*<0.001 by Bonferroni test.

An increase in blood vessel density was found in the PBS-treated group animals, indicating that angiogenesis is a normal response to the ischemic injury. However, cell transplantation induced a significative increase in blood vessel density (hMSCs: 5.37%±0.09, 5.61%±0.29, 5.87%±0.16, 5.42%±0.36; and hMAPCs: 5.93%±0.3, 6%±0.61, 6.76±1.2 and 6.02%±0.48, at 2 days, 4 days, 7 days and 28 days respectively) compared to the PBS-treated group of mice (5.06%±0.22%, 5.33%±0.15, 5.7±0.06% and 5.14±0.25%), *p*<0.05 and *p*<0.001. Importantly, hMAPCs produced a significant higher effect on neovascularization than hMSCS, *p*<0.001 (Fig. 2A–B), suggesting that hMAPC exert a more robust effect on neovascularization.

### Transplantation of hMAPCs and hMSCs is Associated with Attenuation of the Inflammatory Response

**Figure 3:**
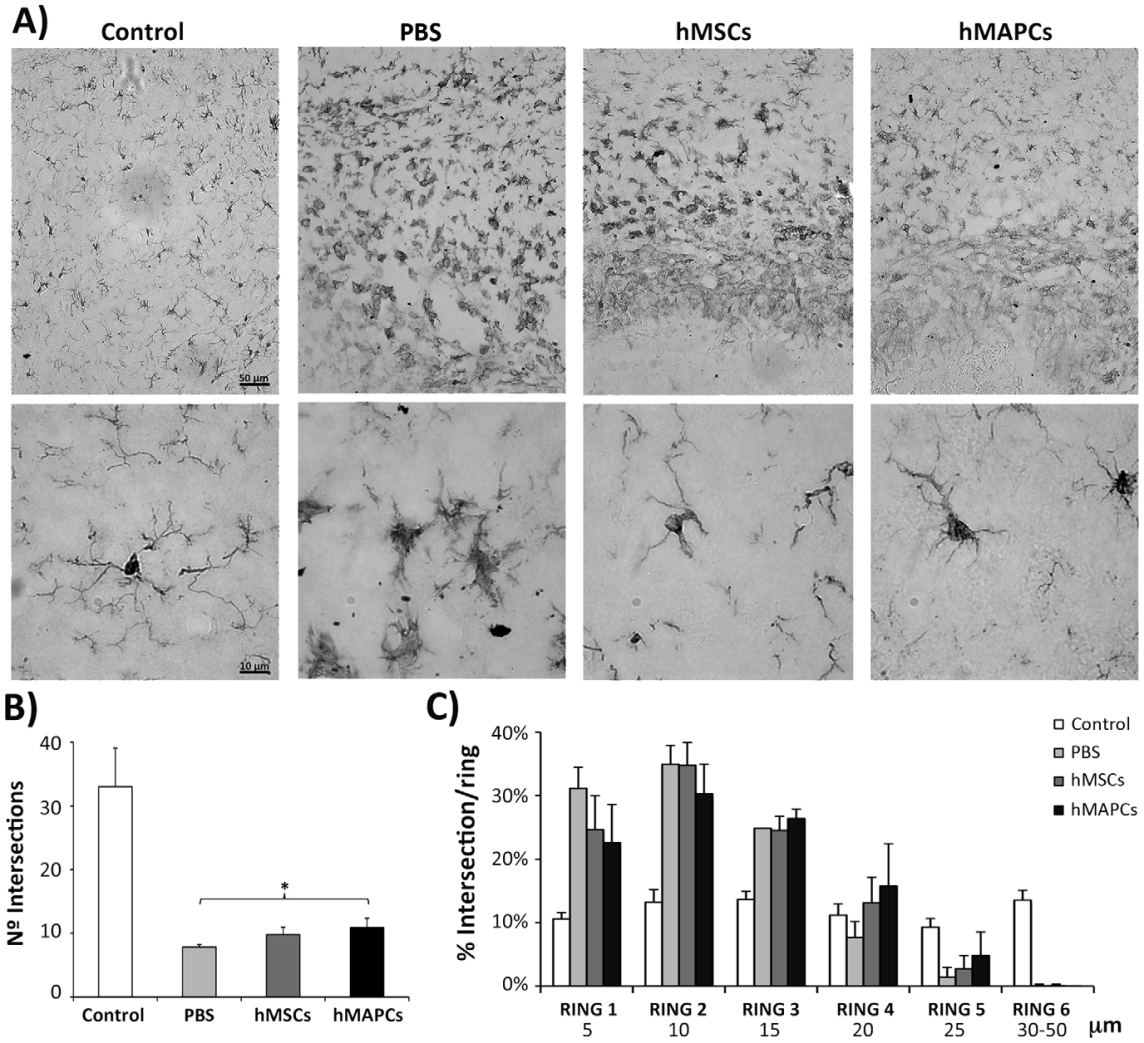
Effect of hMSCs and hMAPCs transplantation on microglia activation after stroke. A) Immunostaining with anti-Iba1 in sections corresponding to the peri-infarct zone 2 days after cell transplantation. Upper panel represents a panoramic view (Scale bar = 50 µm) while a detail image is provided in the lower panel (Scale bar = 10 µm). B) Quantification of the mean number of intersections of microglial processes/cell with the grid. C) Representation of the percentage of intersections with each concentric circle of the grid/cell. *p<0.05 for PBS-treated group vs. hMAPCs by Bonferroni test.

An inflammatory response in the brain is associated with changes in the shape of microglia. Under normal conditions, microglia show a ramified appearance with a large number of processes. In inflamed brain, microglia acquire a rounded amoeboid shape and the length and number of processes is decreased [41]. To determine the effect of cell transplantation on the inflammatory response, immunostaining against Iba1 was performed 4 days after cell transplantation. Qualitatively, a decrease in the number and the grade of activation (reduced somata size and increase in the ramification of the cytoplasmic processes) was observed after cell transplantation compared to the PBS-treated group of mice (Fig. 3A).

To quantify the effects of hMSCs and hMAPCs on microglial activation, the total number of intersections and the percentage of intersections per ring were estimated with a grid of concentric circles, 5 µm apart. Although an increase in the number of intersections was found after treatment with hMSCs and hMAPCs (9.78±1.18 and 10.88±1.47, respectively) in comparison with the PBS-treated mice (7.86±0.35) (Fig. 3B), these differences with PBS treated mice only reached significance for hMAPC treated mice (*p*<0.05). Further, in PBS-treated animals a higher percentage of intersections per ring in the first and second ring were found (shorter processes), while in animals treated with hMSCs more intersections were present in the second and third ring; and the intersections in animals treated with hMAPCs were mostly found in the distal rings (Fig. 3C). These results suggest a more pronounced attenuation of the inflammatory response of hMAPCs in comparison to hMSCs or PBS.

**Figure 4:**
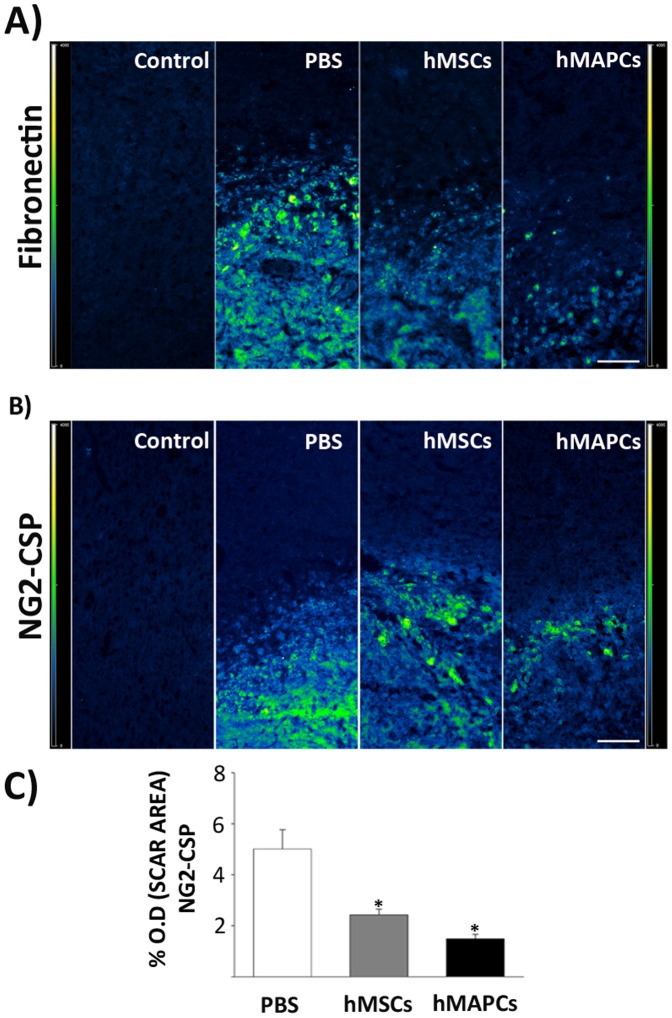
Glial-scar inhibitory effect after hMSCs and hMAPCs transplantation. Immunoflurescence analysis of fibronectin (A) and NG2-CSP (B) 4 days after hMSCs and hMAPCs transplantation. The intensity of the staining varied according to the color scale shown in the left corner in each panel. C) Quantitative analysis of NG2-CSP immunostaining. *P<0.01 in PBS-treated group vs cell transplanted groups. Scale bar = 100 µm.

As transplantation of hMSCs in stroke models has been associated not only with a decrease in inflammation, but also reduced scarring [5], [15], we analyzed the effect of cell transplantation on scar formation by immunostaining against fibronectin and NG2-CSP. A decrease in expression of both proteins was observed 4 days after cell transplantation (*p*<0.01) (Fig. 4), indicating the potential of hMAPCs and hMSCs to provide a neuroprotective effect, minimizing neuronal injury and glial scar formation.

### Transplantation of hMAPCs and hMSC is Associated with an Increase in Endogenous Regeneration due to SVZ Proliferation

Ischemic stroke triggers increased neuroprecursor cell proliferation in the SVZ and migration of these newly formed neuroblasts towards the damaged area, in an attempt to self-repair after the ischemic insult [42], [43], [44].

**Figure 5:**
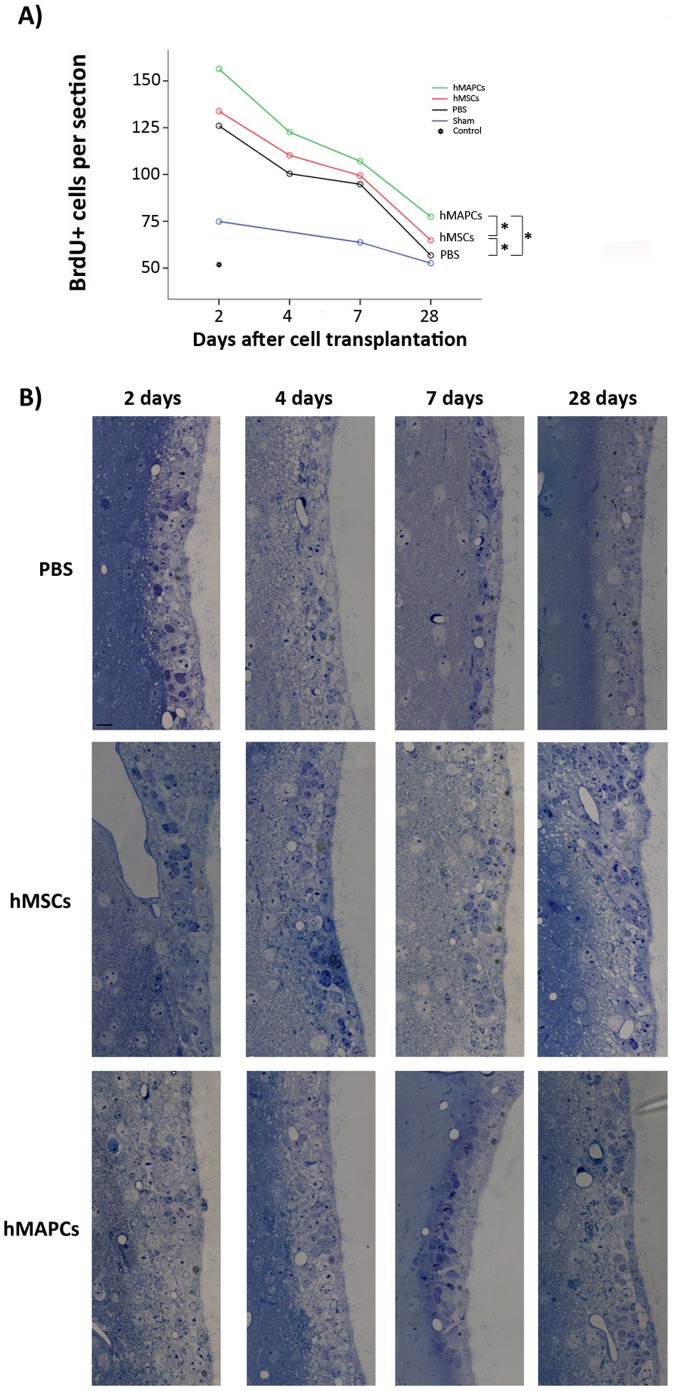
Endogenous neural stem cell activation after hMSCs and hMAPCs transplantation. A) Quantification of BrdU+ cells in the SVZ at different time points (2 days, 4 days, 7 days and 28 days) after hMSCs and hMAPCs transplantation. Cells were counted in the ipsilateral side of the ischemic brains and in non-ischemic brains (control). *p<0.05 by Bonferroni test. B) Sections corresponding to the upper third of the SVZ that shows the degree of proliferation of the SVZ at different time points after PBS, hMSCs and hMAPCs transplantation. Scale bar = 20 µm.

To examine whether cell therapy with hMAPCs or hMSCs could contribute to this effect, a single dose of BrdU was administered at different time points before sacrifice in the 3 groups of mice, and the activation of cell proliferation in the SVZ was quantified. Transplantation of both cell types produced a significant enhancement of proliferation in the SVZ (hMSCs: 133.83±3.82, 110.24±3.69, 99.42±4.26, 64,87±4.52 cells/section; and hMAPCs: 156.46±7.9, 122.67±6.42, 107.08±6.98, 77.46±2 cells/section, at 2 days, 4 days, 7 days and 28 days after cell transplantation respectively) in comparison to PBS-treated mice (126±2.69, 100.42±3.7, 94.83±2.37 and 56.79±4.11 cells/section) (*p*<0.005) (Fig. 5A). Importantly, proliferation was significantly higher in the hMAPC compared with hMSC treated animals, *p*<0.005 (Fig. 5A), suggesting that hMAPCs exert a more robust effect on activation of SVZ proliferation.

Activation of SVZ cell proliferation following cell transplantation was confirmed by the analysis of the upper third of the SVZ in semithin sections. The 2–3 cell layers characteristic of wt mice SVZ increased after stroke and PBS or cell transplantation. Within each group, activation was higher 2 days after cell transplantation, decreasing progressively at latter time points. 2 days after cell transplantation, activation of cell proliferation resulted in an increase of the number of cells as well as in the number of cells layers to 4–5 cells thickness. Qualitatively, mice treated with hMAPCs showed a greater activation of SVZ proliferation than animals treated with hMSCs or PBS as the number of cells and cell layers is higher at all time points analyzed (2 days, 4 days, 7 days and 28 days after PBS or cell transplantation (Fig. 5B). Activation of cell proliferation decreases progressively with time, decreasing the number of cells and cell layers in the SVZ. However, at all the time points analyzed the number of cell and cells layers is higher when stem cells are transplanted compared to PBS, and more pronounced for hMAPCs. These results are in agreement with the quantification of BrdU+ cells in the SVZ.

**Figure 6:**
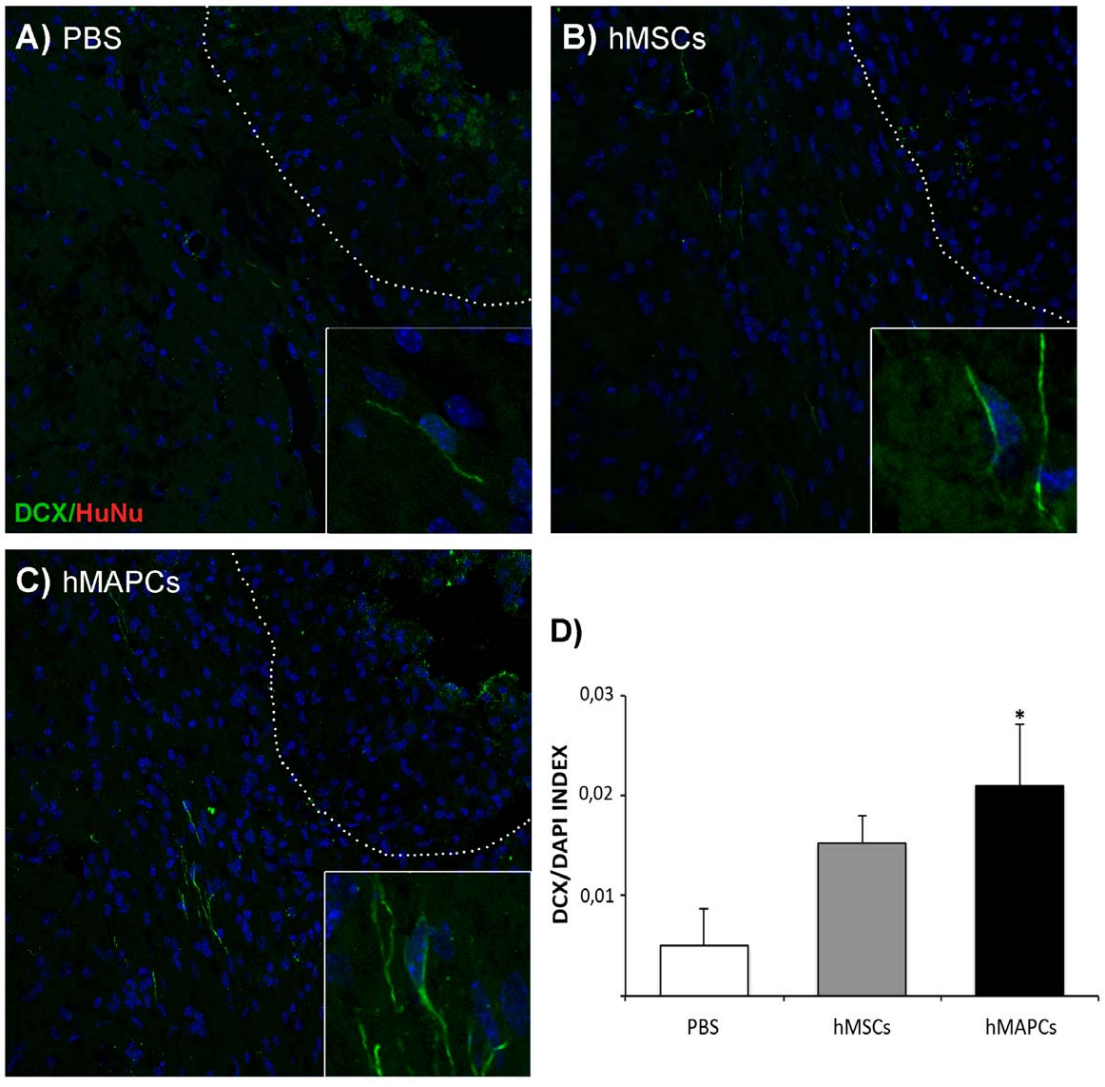
Endogenous neuroblast survival. A–C) Double immunostaining against DCX (green), HuNu (red) and DAPI (blue) in the peri-infarct zone 28 days after cell tranplantation. Scale bar = 50 µm. Magnification scale bar = 75 µm. D) Representation of the DCX/DAPI index estimated in the scar boundary zone 28 days after hMSCs and hMAPCs transplantation. *p<0.05 for PBS-treated group vs hMAPCs by Bonferroni test.

Finally, to determine whether transplantation of hMSCs and hMAPCs promotes migration and survival of endogenous cells in the ischemic brain, double immunostaining against DCX (a marker of immature or early neurons) and HuNu (to identify transplanted cells) was performed 28 days after transplantation. As previously described no transplanted cells were detected after 28 days. On the other hand, an increase in the number of DCX positive cells was found in the scar boundary zone in animals treated with hMAPCs and hMSCs in comparison to PBS-treated mice (Fig. 6A–C). Estimation of the DCX/DAPI index confirmed the increase of DCX^+^ cells (PBS: 0.005±0.003; hMSCs: 0.015±0.003 and hMAPCs: 0.021±0.006). However, this difference only reached significance when we compared hMAPCs to PBS-treated animals (*p*<0.05) (Fig. 6D). Interestingly, hMAPCs appear to induce more neuroblast survival in the scar boundary zone raising the possibility of additional neurogenic effects.

## Discussion

Transplantation of MSCs has been successfully used for the treatment of experimental stroke paving the way for the initial clinical studies in patients with ischemic stroke [22], [23], [24]. Administration of MSCs either intravenously or directly in the periphery of the infarct induces a functional benefit that is associated with pleiotropic effects including the enhancement of neurogenesis and angiogenesis, as well as, positive effects on glial induced scarring and axonal regeneration [1], [11], [14], [15], [16], [17]. The direct comparison of hMSC with a new population of bone marrow derived cells, hMAPCs, indicates that the latter compared favorably with hMSC and provides a greater beneficial effect as indicated by the increase in angiogenesis, SVZ cell proliferation and decreased inflammatory response providing an attractive new source of allogeneic source of cells for stroke.

In order to examine clinical grade cell therapy products, we chose an immunodeficient mouse model (BALB/Ca-RAG 2^−/−^ γC^−/−^) that allowed us to transplant human cells [45] in an immune permissive milieu, avoiding cell rejection, enabling transplanted cell to survive longer and elicit their beneficial effects via the release of trophic factors that enhance the recovery. The lack of a normal inflammatory response in these animals could be considered a limitation of our model, as not only local inflammatory cells (glial cells) but also systemic cells participate in the healing process after stroke. Nevertheless, the use of the PBS-treated group of animals (injected with PBS alone) supports our observations regarding hMAPCs and hMSCs as capable of providing a neurorestorative effect after stroke.

hMSCs and hMAPCs engrafted short term and migrated from the site of transplantation to the injured tissue as early as 4 days after cell transplantation, surviving for at least 14 days post-injection. It is known that SDF-1/CXCR4 plays a role in mediating hMSCs migration to damaged tissue, given that SDF-1 becomes up-regulated in the ischemic boundary zone and hMSCs express its receptor CXCR4 [17]. We postulate that hMAPCs also express CXCR4 and migrate in a similar way, as this mechanism has been described not only for MSCs, but also for NSCs and EPCs [3].

In this study human cells disappeared progressively and were no longer detected 4 weeks after transplantation, in contrast to other studies where transplanted cells persisted longer. These differences could be due to the cell type used (embryonic cells, NSCs, MSCs, MAPCs…), the method of administration, the total number transplanted cells (2×10^5^–2×10^6^) and, most interestingly, the time point of administration. As we grafted the cells early after stroke, significantly more inflammation was present compared with later time points following stroke, constituting a more hostile environment for the transplanted cells [4], [17], [46], [47], [48]. We purposefully grafted the cells on day 2, to impact most profoundly on the inflammation. However, the micro environmental inflammatory-ischemic conditions may explain why the transplanted cells disappeared.

The issues that need to be solved before moving to the clinical application of cell therapy in ischemic stroke have been recently discussed and constitute the road map for the STEPS consortium [7], [49]. Among these issues, the mechanism contributing to neuroprotection and/or neuroregeneration is not fully understood. One of the major therapeutic effects of cell therapy in stroke is the support of the cells to angiogenesis, in part due to the upregulation of angiogenic growth factors and receptors, such as VEGF, VEGFR and angiopoietins [5]. In the current study, we did not examine the production of angiogenic growth factors from MSCs or MAPCs but as previously shown, both human and mouse MAPCs produce significant amounts of VEGF, PlGF, bFGF, PDGF-BB and even TGF-β, all of which are implicated in angio-vasculogenesis [50], [51]. We hypothesize that higher levels of these growth factors in hMAPCs versus hMSCs could explain the greater effect observed in animals treated with hMAPCs.

Another potential repair mechanism is the ability of hMSCs and hMAPCs to attenuate the robust inflammatory response, which takes place within hours after stroke and represents a key factor for brain damage. This idea is supported by the analyses of microglial activation 4 days after cell transplantation, which demonstrated a significant reduction in the degree of activation. Interestingly, it has been demonstrated that rMAPCs release anti-inflammatory cytokines, such as IL-4 and IL-10, that regulate microglia *in vitro* [52], and thus could be responsible for the reduced activation of microglia in mice treated with hMAPCs in comparison with hMSCs. MSCs have been described to substantially reduce expression of axonal-growth inhibitory proteins, such as reticulon (Rtn4; also known as Nogo), enabling axonal and neurite outgrowth [53] both *in vitro* and *in vivo*, so it is tempting to speculate that transplantation of MAPCs is associated with similar effects contributing to axonal regeneration. The reduction in the scar and the marked decrease in both NG2-CSP and fibronectin expression could provide a more permissive environment, further promoting axonal sprouting with new connections being established, eventually leading to improvement in neurological function.

Stroke promotes cell proliferation in the SVZ and increases the number of immature neurons that migrate into the severely damaged area [42], [44]. However, the vast majority of these cells usually die. This degeneration probably reflects the detrimental effects of the severely damaged tissue, which lacks appropriate trophic support and connections. Our results demonstrate that hMSCs and hMAPCs enhanced SVZ proliferation and neuroblast survival by mechanisms likely related to the capacity of the cells to act as small biological pumps that secrete neurotrophins (BDNF), cytokines, and growth factors (VEGF, bFGF, NGF) with autocrine, as well as paracrine effects, on resident cells [54]. It is important to point out that neurogenesis and angiogenesis are highly interdependent processes. Activated endothelial cells secrete many factors such as stromal-derived factor 1α and matrix metalloproteinases (MMPs) that mediate neuroblast migration and survival, attracting neuroblasts from the SVZ and probably into a lesser extent from the striatal parenchyma (dormant neurons or latent progenitors) to the boundary zone of the ischemic lesion while the release of MMPs allows for the degradation of the extracellular matrix. In addition to guiding neuroblast migration, activated endothelial cells secrete VEGF that increases neurogenesis [47]. The fact that hMAPCs induced an increase in angiogenesis in comparison with hMSCs could also explain the greater effect on SVZ proliferation and neuroblast survival.

### Conclusions

In summary, we demonstrate that transplantation of hMSCs and hMAPCs mediate neuroprotection during the acute phase of ischemic stroke and prevents delayed brain injury through several therapeutic effects, such as angiogenesis, diminished inflammation and scarring, as well as, increased SVZ cell proliferation and neuroblast survival close to the ischemic area. Therapeutic effects mediated by cell transplantation do not require long-term cell engraftment and are likely related to the secretion of growth factors and cytokines. Experimental data suggest that hMAPCs clone (B30/E2) may produce greater therapeutic effects in comparison with hMSCs clone (JN2) and raising the possibility to use hMAPCs as an alternative source of cells for transplantation in patients suffering from stroke given their therapeutic potential and the possibility of producing them under GMP conditions. However, prior to making these assumptions more clones of hMSCs and hMAPCs should be compared.

## Supporting Information

Figure S1 **Characterization of hMSCs and hMAPCs by flow cytometry.** Cells from MAPC clone B30A2 and MSC clone JN1 were stained with antibodies against CD44, CD45, HLA-II, CD144, CD34, HLA-I, CD105, CD140a, CD90, CD73, CD117, and CD31 (blue line) or isotype controls (black line).
(TIF)

Figure S2 **Experimental design.** Diagram with the groups, animal per group and time points analyzed.
(TIF)

Figure S3 **hMSCs and hMAPCs survival and migration.** Anti-human nuclei immunostaining to detect human cells after 2 days and 7 days of transplantation. A–B higher magnification pictures of depicted squares in the panoramic views. Panoramic view scale bar = 500 µm. Magnification scale bar = 100 µm.
(TIF)
