# Supplementary figures and images for: Therapeutic Effects of hMAPC and hMSC Transplantation after Stroke in Mice
Source: PLoS One. 2012 Aug 31;7(8):e43683. doi: 10.1371/journal.pone.0043683 (PMC3432058; doi:10.1371/journal.pone.0043683)

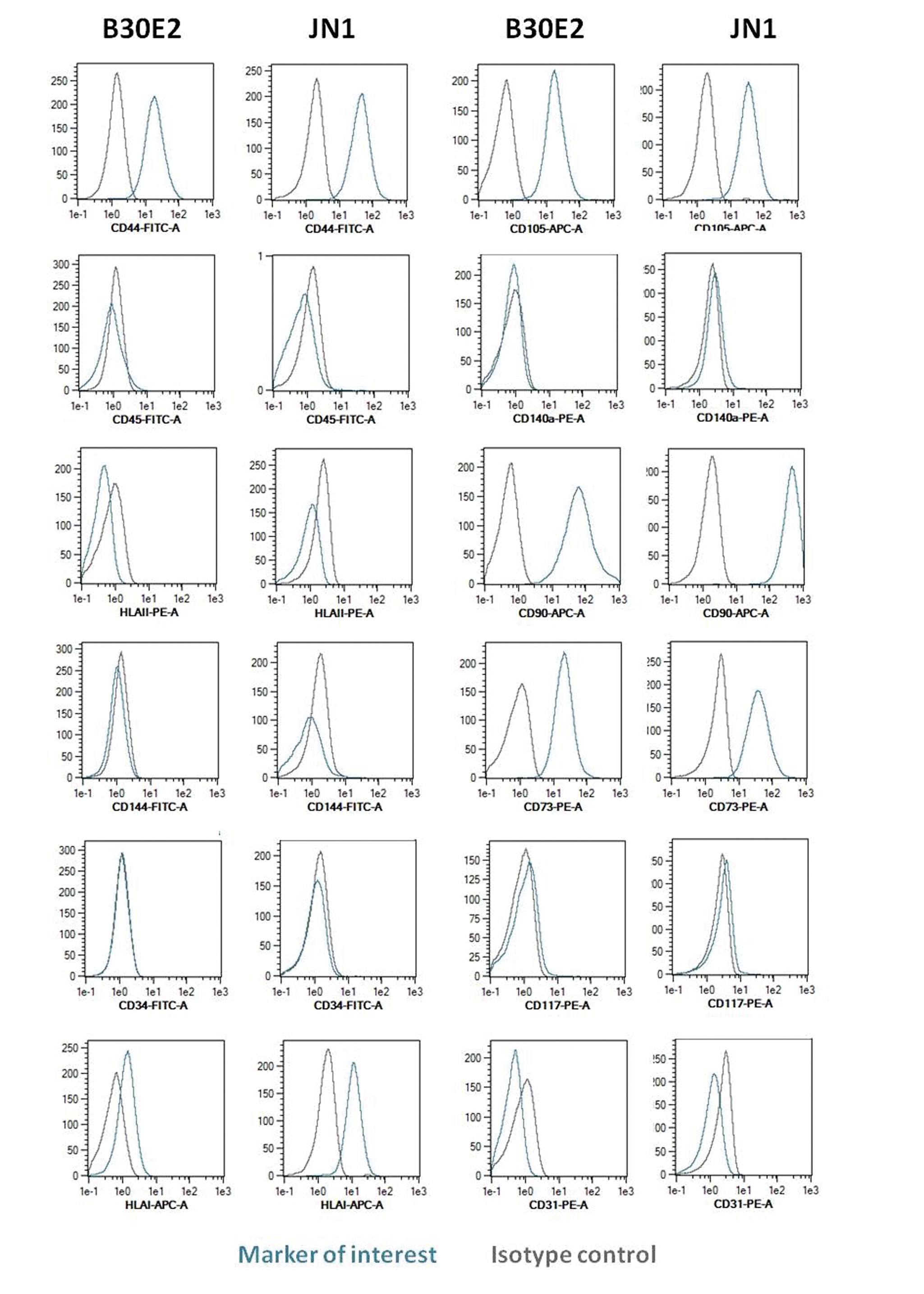

Supplement: Figure S1 — Characterization of hMSCs and hMAPCs by flow cytometry. Cells from MAPC clone B30A2 and MSC clone JN1 were stained with antibodies against CD44, CD45, HLA-II, CD144, CD34, HLA-I, CD105, CD140a, CD90, CD73, CD117, and CD31 (blue line) or isotype controls (black line). (TIF) [file pone.0043683.s001.tif]

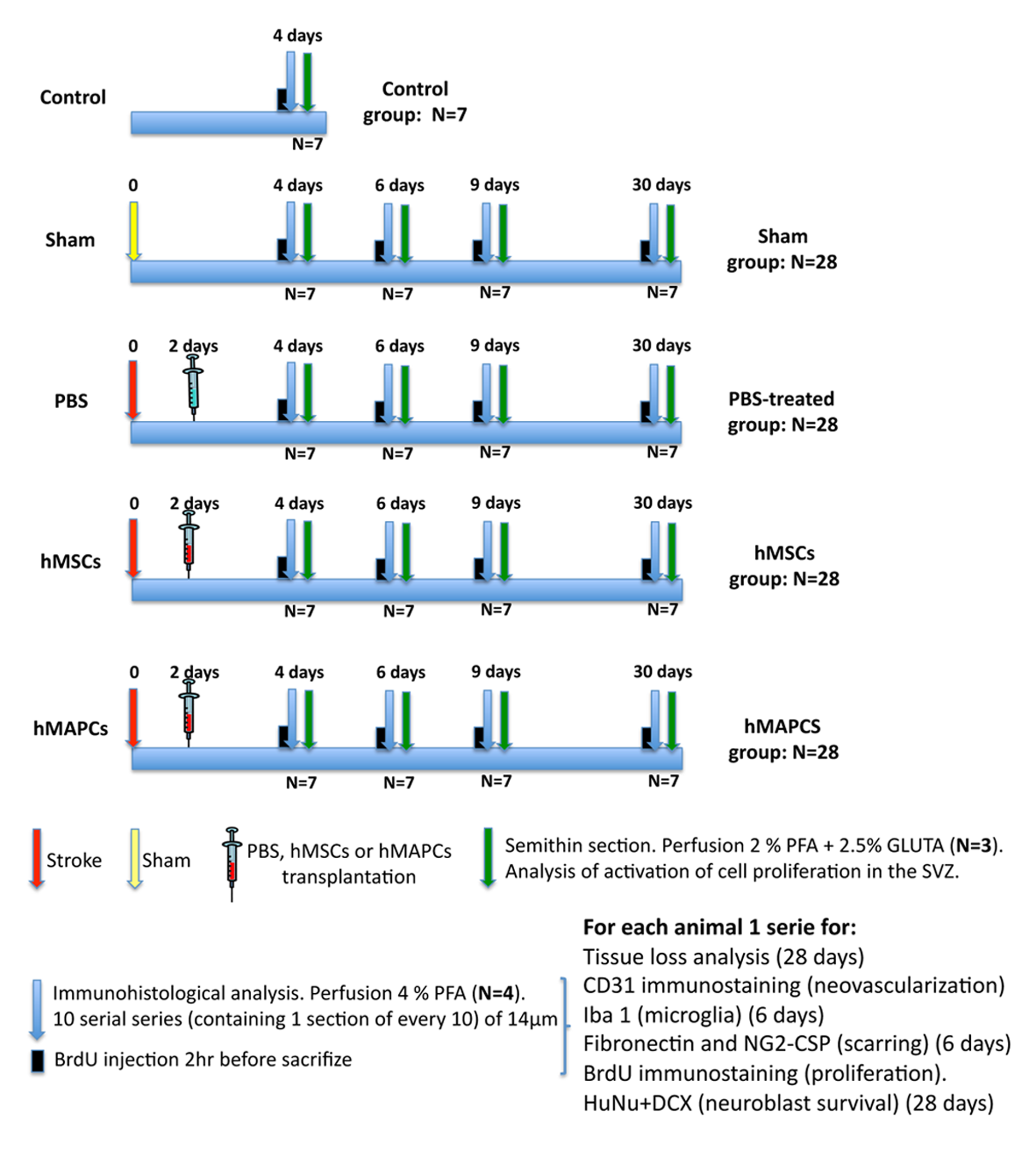

Supplement: Figure S2 — Experimental design. Diagram with the groups, animal per group and time points analyzed. (TIF) [file pone.0043683.s002.tif]

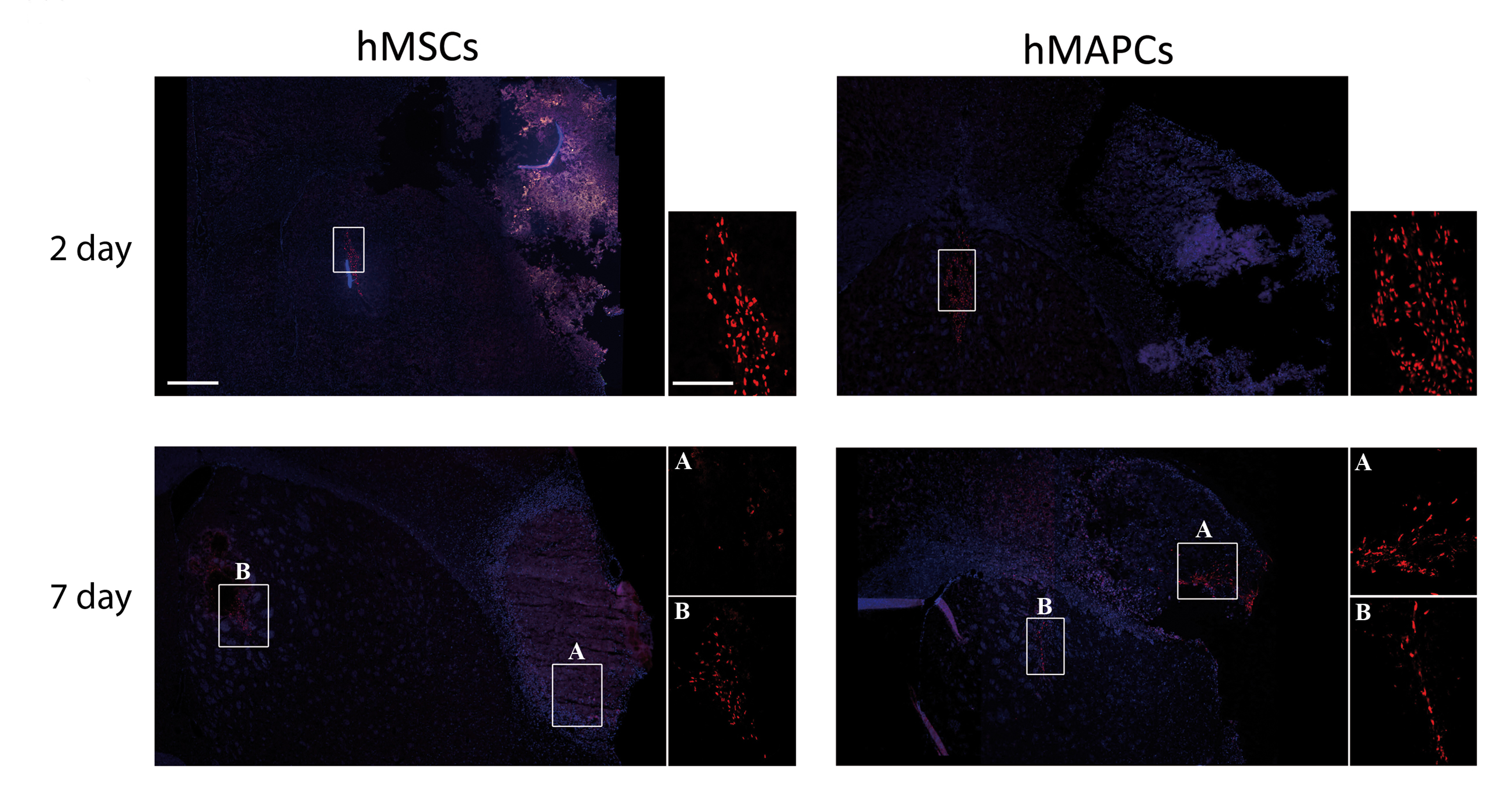

Supplement: Figure S3 — hMSCs and hMAPCs survival and migration. Anti-human nuclei immunostaining to detect human cells after 2 days and 7 days of transplantation. A–B higher magnification pictures of depicted squares in the panoramic views. Panoramic view scale bar = 500 µm. Magnification scale bar = 100 µm. (TIF) [file pone.0043683.s003.tif]
